# On the Other Side: Manipulating the Immune Checkpoint Landscape of Dendritic Cells to Enhance Cancer Immunotherapy

**DOI:** 10.3389/fonc.2019.00050

**Published:** 2019-02-06

**Authors:** Benjamin Y. Kong, Holly Bolton, Julius W. Kim, Pablo A. Silveira, Phillip D. Fromm, Georgina J. Clark

**Affiliations:** ^1^Dendritic Cell Research Group, ANZAC Research Institute, Concord, NSW, Australia; ^2^Sydney Medical School, The University of Sydney, Camperdown, NSW, Australia; ^3^Department of Medical Oncology, Concord Repatriation General Hospital, Concord, NSW, Australia

**Keywords:** dendritic cells, immune checkpoints, PD-1, CTLA-4, immunotherapy

## Abstract

Monoclonal antibodies targeting co-inhibitory immune checkpoint molecules have been successful in clinical trials of both solid and hematological malignancies as acknowledged by the 2018 Nobel Prize in Medicine, however improving clinical response rates is now key to expanding their efficacy in areas of unmet medical need. Antibodies to checkpoint inhibitors target molecules on either T cells or tumor cells to stimulate T cells or remove tumor mediated immunosuppression, respectively. However, many of the well-characterized T cell immune checkpoint receptors have their ligands on antigen presenting cells or exert direct effects on those cells. Dendritic cells are the most powerful antigen presenting cells; they possess the ability to elicit antigen-specific responses and have important roles in regulation of immune tolerance. Despite their theoretical benefits in cancer immunotherapy, the translation of DC therapies into the clinic is yet to be fully realized and combining DC-based immunotherapy with immune checkpoint inhibitors is an attractive strategy. This combination takes advantage of the antigen presenting capability of DC to maximize specific immune responses to tumor antigens whilst removing tumor-associated immune inhibitory mechanisms with immune checkpoint inhibition. Here we review the expression and functional effects of immune checkpoint molecules on DC and identify rational combinations for DC vaccination to enhance antigen-specific T cell responses, cytokine production, and promotion of long-lasting immunological memory.

## Introduction

**Figure 1:**
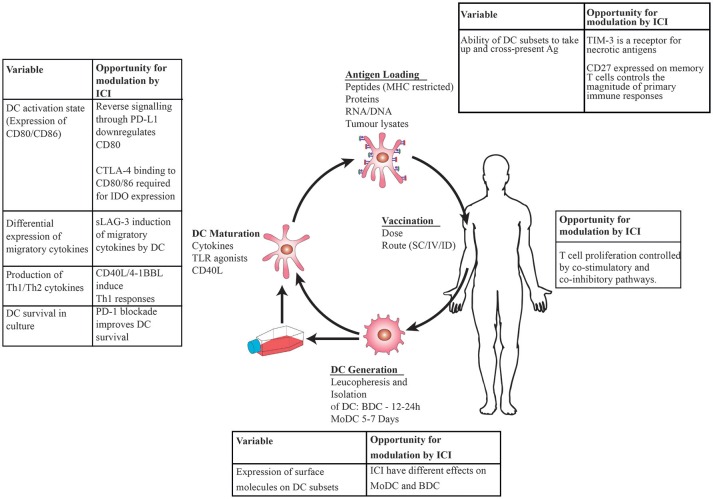
Schematic showing DC vaccination: DC are generated by leucopheresis and isolation by immunoselection, matured *ex vivo* using cytokines then loaded with tumor antigens prior to injection back into the patient. Immune checkpoint inhibitors (ICI) administered at the time of DC maturation and antigen loading will have direct effects on DC in addition to modulating T cell: tumor interactions, leading to opportunities to modulate immune responses at the level of DC, T cell interactions.

Dendritic cells (DC) are key components of the immune system which control innate and adaptive immunity, inducing clonal selection of T cells, eradication of infection, and tolerance to self (1). Their ability to prime naïve T cells and induce antigen-specific T-cell memory makes them an attractive target for anti-cancer immunotherapy. DC vaccination is the process by which DC are isolated from patients, loaded with tumor antigens, matured *ex vivo*, and subsequently re-administered systemically (Figure 1). It exploits the ability of DC to process and present foreign antigens, mature to enable optimum T cell stimulation and migrate to lymphoid organs where naïve T cells are encountered and turned into effector cells (2).

Despite the potential benefits of DC vaccines, to date they have shown minimal overall survival benefit in clinical trials as monotherapy. Sipuleucel-T, the first FDA-approved cellular cancer vaccine (3), has been followed by other phase III DC vaccine trials. This includes Rocapuldencel-T (NCT01582672) for renal cell carcinoma (RCC) and a similar vaccine for melanoma (4), both of which were ceased prematurely due to poor efficacy. The trial of Rocapuldencel-T included patients with previously untreated intermediate or high risk metastatic RCC (5) who were treated with sunitinib alone in the control arm with the DC vaccine added to the experimental arm. The selection of intermediate and high risk patients as well as subsequent improvements in systemic treatment (6) mean that overall survival is expected to be better than if more favorable prognostic groups or current systemic treatments were used as a control arm. Therefore, it is likely that the lack of survival benefit from DC vaccination is due to inherently low efficacy rather than trial design. An ongoing phase III trial using the DC-Vax™ platform for glioblastoma multiforme (NCT00045968) recently reported encouraging interim overall survival results (7) for which mature data reporting unblinded treatment groups are awaited.

Variations in *ex vivo* preparation of DC provide some explanation for this lack of efficacy. These variations, addressed in a recent review (8), include the choice of DC, degree of DC maturation, route of administration, and choice of target antigen. The challenge of identifying reasons for trial failure is illustrated by the heterogeneity of preparations used in key phase III trials. Sipuleucel-T is manufactured by density gradient enrichment of peripheral blood mononuclear cells (PBMC) loaded with prostatic acid phosphatase (PAP) peptide fused to GM-CSF (9), whilst Rocapuldencel-T is manufactured with monocyte-derived dendritic cells (MoDC) loaded with tumor neo-antigens in the form of mRNA (10). Lastly, the DC-Vax™ platform consists of MoDC pulsed with patient-derived tumor lysates. All these differences are likely to result in vast differences in the ability of DC to induce effector and memory T cell responses *in vivo*. Another explanation is the ability of tumor cells to create an immunosuppressive microenvironment by expression of ligands for T cell co-inhibitory molecules, many of which are also expressed to varying degrees on DC.

**Figure 2:**
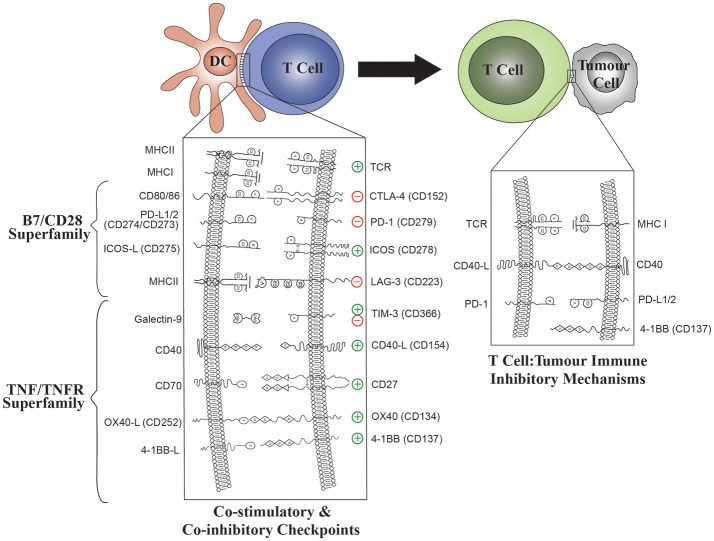
The role of B7/CD28 and TNFR Superfamily co-stimulatory and co-inhibitory molecules in both DC: T-cell and T cell: tumor interactions. Modulation of ligand expression on DC will enable optimal T cell stimulation. The effect of DC ligands on T cell effector functions is shown for each ligand: receptor pair.

Immune checkpoints are immune regulatory pathways which begin with interactions between receptor ligand pairing of molecules on the cell surface that modulate self-tolerance and control the amplitude of immune responses in peripheral tissues (11) (Figure 2). Blockade of T cell co-inhibitory molecules such as CTLA-4 and PD-1 with monoclonal antibodies (mAb) (“immune checkpoint inhibitors”) to remove tumor-associated immune tolerance has been successful in clinical trials in cancer (12–14). Administration of immune checkpoint inhibitors will result in binding to molecules expressed on DC as well as T cells. Despite the presence of many of the same receptors or their ligands on DC (Figure 2), modulation of these pathways in DC has been overlooked as a rationale for clinical trials to date.

The “tidal” model of an immune response relies on a dynamic environment of spatiotemporal interactions between ligands and receptors in addition to static expression patterns of ligand-receptor pairs (15). Nevertheless, the expression patterns of individual ligand-receptor pairs and the *in vitro* functional consequences provide an insight into the physiological roles.

DC vaccination in combination with immune checkpoint inhibitors is a rational step which addresses the clinical problem of primary or acquired resistance (16) to immune checkpoint blockade. DC have the potential to turn immunologically “cold” tumors into “hot” tumors (17) by several different mechanisms. Activation of pathways such as the STING pathway, a key link between the innate and adaptive immune systems, promotes production of pro-inflammatory cytokines by DC (18) and alteration of the tumor microenvironment. The efficacy of immune checkpoint inhibitors in tumors with a high mutational burden (19) has led to the use of DC loaded with tumor neoantigens (NCT03300843) in a bid to stimulate immune responses and broaden the immunogenicity of some tumors. Increasing tumor mutational burden correlates well with the lymphocytic infiltrate seen in tumors. In addition to removal of tumor-associated immunosuppression toward tumor-specific infiltrating lymphocytes immune checkpoint inhibitors also act directly to enhance DC production of Th1 polarizing cytokines, augment antigen-specific priming of naïve T cells and promote long-lasting T cell memory (20–23). *Ex vivo* DC vaccination affords the opportunity to stimulate expression of immune checkpoint receptor ligands on DC during the maturation process to orchestrate T cell responses.

A deeper understanding of the functional role of immune checkpoint ligands and receptors on DC is needed to define the role of combination therapies and translate DC therapies into the clinic. Here we review the literature on the expression and function of B7/CD28 and TNF/TNFRSF immune receptor superfamilies on DC, focusing on molecules currently in clinical use to identify rational combinations for translation of DC vaccination combined with immune checkpoint inhibitors.

## The B7/CD28 Family

Co-stimulation of T cells occurs following T cell receptor (TCR) engagement by antigen bound to MHC molecules on APC such as DC (Figure 2) (24). The B7/CD28 family, a cluster of immunoglobulin superfamily receptors, provides co-stimulatory or co-inhibitory signals following TCR engagement (Figure 2) (25). B7 family ligands such as CD80 or CD86 are typically expressed on APC whilst CD28 receptors are expressed on T cells. Interaction of CD80/86 with CD28 stimulates naïve T cell responses that are attenuated by the negative regulator, CTLA-4 (CD152). This is one of the earliest and most potent co-stimulatory events, as evidenced by the fatal autoimmune phenotype of CTLA-4 knockout mice (26). The CD28 receptor may be activated directly by superagonists such as the TGN1412 mAb, resulting in a catastrophic cytokine storm when administered systemically to humans (27, 28). The function of CTLA-4 is illustrated clinically by the milder autoimmune toxicities of anti-PD-1 antibodies such nivolumab or pembrolizumab compared with anti-CTLA-4 antibodies such as ipilimumab (13, 29). To date, blockade of co-inhibitory pathways like CTLA-4 and PD-1 with mAbs have been successful in clinical trials. However, in addition to targeting co-inhibitory pathways to remove tumor-associated immune suppression, there is strong interest in targeting co-stimulatory molecules such as ICOS in an attempt to prolong T cell responses and promote desirable effects such as immunological memory (23, 30, 31). This strategy is being used, amongst other reasons, to address the clinical problem of acquired resistance to PD-1 blockade. Other B7/CD28 family receptors, such as ICOS and the immunoglobulin superfamily members, TIM-3 and LAG-3, are associated with later events in T cell co-stimulation (15).

### Cytotoxic T Lymphocyte-Associated Protein-4 (CTLA-4, CD152)

CTLA-4 is a co-inhibitory receptor with a high degree of sequence similarity to the T cell co-stimulatory receptor, CD28. Blockade of CTLA-4 enhances CD4+ effector T cell activity (32) whilst CTLA-4 expression on Tregs exerts a cell-extrinsic immunosuppressive effect by downregulating CD80 and CD86, the CD28 ligands which are expressed on DC (33) (Figure 2). The ability of CTLA-4 blocking mAbs to enhance anti-tumor immunity in preclinical models (34) has led to their use in the treatment of metastatic melanoma (29).

CTLA-4 is expressed on activated T cells in humans (35). CTLA-4 blockade with mAbs prevents binding of CD80 and CD86 on DC to CTLA-4 on T cells. T cell associated CTLA-4 binding to CD80 or CD86 is required for expression of the immunosuppressive enzyme indoleamine 2,3-dioxygenase (IDO) in DC *in vitro* (36). IDO exerts an immunosuppressive effect on T cell proliferation, therefore blockade of CTLA-4 is expected to have a positive effect on T cell proliferation via its action on DC. This mechanism has not been verified *in vivo* with naturally circulating blood dendritic cells (BDC). The cell-intrinsic effect of CTLA-4 blockade on effector T cells has been shown in mice and humans to be the primary mechanism for the anti-tumor effect, rather than its effect on Treg cells (37, 38). The use of an anti-CTLA-4 mAb (tremelimumab or ipilimumab) to treat prostate cancer and melanoma patients results in broadening of the T cell repertoire (39), in keeping with this mechanism of action.

CTLA-4 expressed on MoDC has an emerging immune regulatory role (40). Binding of this receptor by an activating CTLA-4 mAb has an inhibitory effect, enhancing IL-10 secretion and decreasing T cell proliferation. Furthermore, CTLA-4 is secreted and B7 molecules downregulated on bystander MoDC following cytokine stimulation (41).

Combination of anti-CTLA-4 mAbs with DC vaccination is predicted to remove the inhibitory effect of CTLA-4 ligation on DC and act in synergy with the stimulatory effects of CD80 and CD86 which are upregulated by DC maturation. Clinical studies such as the combination of DC pulsed with the tumor antigen NY-ESO with or without CTLA-4 blockade (NCT02070406) will answer the question on the relevance of this mechanism *in vivo*.

### Programmed Cell Death 1 Protein (PD-1, CD279)

PD-1 was described early on as a marker of programmed cell death in lymphocytes playing a role in limiting inappropriate immune responses (42). When bound by its ligands, PD-L1 (B7-H1/CD274) or PD-L2 (B7-DC/CD273), PD-1 transmits an inhibitory signal in the presence of simultaneous TCR engagement, resulting in inhibition of T cell proliferation and cytokine secretion (IFN-γ, IL-10) (42, 43). The number of PD-1 molecules available to bind its ligands, PD-L1 and PD-L2 determines cytokine production. Ligation of PD-L2 alone inhibits T cell cytokine production to a lesser extent than PD-L1 (43) however selective blocking of PD-L2 is yet to be studied extensively *in vivo*. Blockade of PD-1 restores the capacity of CD8+ T cells to attack tumors in the presence of inhibitory signals from PD-L1 expressed on tumor cells (44). This has led to the successful use of anti-PD-1 and anti-PD-L1 antibodies for the treatment of melanoma, non-small-cell lung cancer, and renal cell carcinoma (12–14).

PD-1 is expressed on a diverse range of lymphoid and myeloid cells (42, 43, 45). In humans, PD-1 exhibits activation-induced expression on T cells (46). PD-1 expression on DC in mice was more recently described (47). DC from PD-1 knockout mice show increased survival *in vitro* compared with wild-type mice. Adoptive transfer of DC from PD-1 knock-out mice confers protection against infection and improved tumor control in a hepatocellular carcinoma murine model (48). Therefore, absence of PD-1 expression has downstream effects which improve DC survival and function.

PD-L1 and PD-L2, the ligands for PD-1, are also expressed upon activation (43, 49). PD-L1 is expressed on a minority of resting lymphoid cells and most myeloid cells while PD-L2 has a much more restricted expression pattern, limited to activated monocytes and APC. In humans, PD-L1 is weakly expressed on both myeloid and plasmacytoid blood DC and may be upregulated preferentially on CD11c+ myeloid DC in the presence of Poly I:C (50) while PD-L2 is expressed on MoDC (49).

*In vitro* targeting of the PD-L1/PD-1 pathway has been achieved with anti-PD-1 mAbs (51) and PD-L1/L2 with Fab fragments (22) and results in increased T cell proliferation and production of cytokines including IFN-γ and IL-10 in MoDC co-cultures. In contrast to the results of mAb experiments, blockade of PD-L1 and PD-L2 with soluble PD-1 inhibits T cell activation via reverse signaling through DC and leading to a suppressive DC phenotype (52). PD-L1 expressed on DC additionally binds to CD80 which is expressed on T cells as well as its expression on DC (53) and has a negative regulatory function. The outcome of PD-L1 blockade is therefore dependent upon the crosstalk between PD-L1 and CD80 on DC and T cells. The expression of PD-L1 on DC has a dual inhibitory effect via PD-L1:PD-1 and PD-L1:CD80 interactions. The addition of soluble PD-1 would only affect the PD-L1: PD-1 pathway, allowing the inhibitory PD-L1: PD-1 and PD-L1:CD80 pathways to predominate. PD-1 blockade alone is unable to overcome T cell tolerance (54), whilst an anti-PD-L1 mAb specific to the PD-L1:CD80 interaction is able to prevent anergy (55). These findings have important clinical implications in cases of intrinsic or acquired resistance to immune checkpoint inhibitors where immune suppressive mechanisms which rely on DC expression of PD-L1. Whether or not anti-PD-L1 mAbs are more effective than anti-PD-1 mAbs in this setting remains to be seen.

PD-1 blockade and DC vaccination is a logical treatment combination which could augment the well-known T cell mediated anti-tumor effect of anti-PD-1 mAbs (44). If given during the DC maturation process (Figure 1), anti-PD-1 mAbs are predicted to improve DC survival (47) (and therefore antigen presentation) and maturation by blockade of reverse signaling through PD-L1 (52). This will lead to production of the Th1 cytokine IFN-γ, conditions which are favorable for anti-tumor immune responses (22).

Further work is needed to determine whether direct targeting of PD-L2 on DC will promote antigen presentation and T cell priming. Specific mAbs to PD-L2 could preferentially target DC and augment their function in addition to disrupting T cell inhibition by tumor-associated PD-L2 (Table 1).

**Table 1:** Dendritic cell, T cell and tumor-associated expression of B7/CD28 and TNFR superfamily ligands and receptors.

**Ligand(s) on DC**	**Receptor**	**Expression on Tumor Infiltrating Lymphocyte (TILs) or Tumors**	Selected mAbs with FDA approval^*^ or in actively recruiting clinical trials^**#**^	**Beneficial Effects of Combination with Immune Checkpoint Blockade for DC Vaccination**
CD80/86	CTLA-4 (CD152)	• **CTLA-4** expressed by activated T cells (56)	*Anti-CTLA-4* • Ipilimumab^*^ • Tremelimumab^#^	• CTLA-4 expressed on DC has a negative regulatory role (40)
PD-L1 (CD274)PD-L2 (CD273)	PD-1 (CD279)	• **PD-1** and **PD-L1** expressed by TILs and tumor cells (57) • Tumor expression of PD-L2 (58)	*Anti-PD-1* • Nivolumab^*^ • Pembrolizumab^*^ • Cemiplimab^*^Anti-PD-L1 • Durvalumab^*^ • Atezolizumab^*^ • Avelumab^*^	• PD-1 blockade prolongs DC survival (47) • Reverse signaling via PD-L1/PD-L2 inhibits DC activation (52)
ICOS-L (CD275)	ICOS (CD278)	• **ICOS** expressed on TILs in CTLA-4 treated subjects (59)	*Agonist* • GSK3359609^#^ • MEDI-570^#^ • JTX-2011^#^	• Controls CD40L dependent antibody class-switching (60)
Galectin-9	Tim-3 (CD366)	• **Tim-3** expressed in TILs (61)	*Antagonist* • MBG453^#^ • TSR-022^#^	• TIM-3 mediates uptake of necrotic antigens (62) • Increased TNF-α production when bound by galectin-9 (63)
MHC Class II	LAG-3 (CD223)	• **LAG-3** expressed by TILs (64)	*Agonist* BMS-986016^#^ • TSR-033^#^ • REGN3767^#^ • IMP321^#^	• sLAG-3-Ig enhances DC maturation and migratory chemokines (65).
CD40	CD40L	• **CD40** expressed in breast cancer (66), head and neck cancer (67) and melanoma (68) cells	*Agonist* • APX005M • CDX-1140 • JNJ-64457107 R07009789/Selicrelumab	• Licensing of DC to produce IL12p70 (21)
CD70	CD27	• CD27 constitutively expressed constitutively by T cells. • **CD70** expressed in CLL (69)	*Antagonist* • ARGX-110^#^ • SGN-CD70A^#^	• Important for T cell priming and memory responses (31)
OX40L (CD252)	OX40 (CD134)	• **OX40** expressed by tumor infiltrating Tregs (70)	• MEDI6469^#^	• OX40-dependent Treg depletion by myeloid cell Fc receptor dependent ADCC (71) • Preferential induction of CD4+ T cell responses (72)
4-1BBL	4-1BB (CD137)	• 4-1BB inducibly expressed on T cells (73) • **4-1BB** expressed on tumor cells (74)	*Agonist* • PF-05082566/Utomilumab^#^ • BMS-663513/Urelumab^#^	• 4-1BB has anti-tumor effects (75) • Preferential induction of CD8+ T cell responses (75)

*Properties of B7/CD28 and TNSRF pathways which augment DC vaccination directly in addition to re-invigorating T cells*.

### Inducible T-Cell Costimulator (ICOS, CD278)

Inducible T-cell costimulator (ICOS) is a co-stimulatory receptor with sequence homology to CD28 (76). ICOS has similar functions to CD28 in its control of T cell proliferation and IL-10 production, but additionally it controls CD40/CD40L dependent antibody class-switching and therefore immunological memory, properties which are desirable for anti-cancer vaccination.

ICOS is expressed on activated T cells, in contrast to the constitutive expression of CD28 (76). Blockade of its ligand, ICOSL, with ICOS-Ig in allogeneic mixed leucocyte reactions between MoDC and T cells leads to reduced T cell proliferation and helper T cell cytokine production for B cell production of IgG and IgM, thereby demonstrating its co-stimulatory role (77). Reduced T cell proliferation in response to recall antigens indicates that that ICOS-L expression on DC is important for controlling T cell responses.

In human BDC subsets, ICOS-L expression is more strongly induced on plasmacytoid DC (pDC) (BDCA-4/CD304+) than mDC in response to activation with the TLR agonist, poly I:C (50) or IL-3 (78). PDC activated by TLR to upregulate ICOS-L induce production of IL-10 by naïve CD4+ T cells (79). Therefore, augmentation of the co-stimulatory effect of ICOS may have the unwanted effect of inducing IL-10 producing Tregs. Further studies are needed to address the expression of ICOS-L on intra-tumoural DC populations and correlate this to expression on BDC. Tumor associated pDC found in close proximity to ICOS+ Treg in breast cancer (80), liver cancer (81), and ovarian cancer (82) are associated with poorer survival. This is indirect evidence that modulation of the ICOS pathway on peripheral pDC negatively affects tumor-associated immunity.

ICOS potentiates anti-tumor immunity mediated by CTLA-4 blockade in murine models of prostate cancer and melanoma (59). A possible mechanism for this is that CTLA-4 blockade on tumor infiltrating lymphocytes leads to ICOS upregulation allowing binding by ICOS-L expressing cells. Anti-CTLA-4 mAbs mediate Fc-receptor dependent depletion of Tregs in mice (83) but not in humans treated with anti-CTLA-4 mAbs (38). Therefore, concomitant administration of anti-CTLA-4 mAbs and DC vaccination under maturation conditions which promote ICOS-L expression is likely to enhance the effect of anti-CTLA-4 mAbs alone. Induction of ICOS-L on DC and the role the ICOS pathway in antibody class switching implies a role for manipulation of DC to promote memory responses when used as vaccine therapy. The use of *ex vivo* DC maturation factors which preferentially upregulate ICOS-L on mDC over pDC will minimize the potential for production of the Th1 inhibitory cytokine, IL-10.

## Other Immunoglobulin Superfamily Members

### T-Cell Immunoglobulin and Mucin Domain Containing Protein 3 (TIM-3, CD366)

TIM-3 is one of 8 *TIM* gene family receptors, of which only three (TIM-1, TIM-3 and TIM-4) are found in humans (84). The expression of TIM-3 as a marker of T cell exhaustion in association with other tumor-associated T-cell exhaustion markers (e.g., PD-1, CTLA-4) (85) has led to its investigation as a target for cancer immunotherapy. However, the characterization of TIM-3 as a T cell exhaustion marker does not take into account its wide expression pattern on other leucocytes such as DC where it is responsible for other immunological effects.

In humans, TIM-3 is expressed on Th1-polarized CD4+ and CD8+ cytotoxic T cells (Tc1) where it acts as a negative regulator of Th1 responses (86). It is a pattern recognition receptor for phosphatidylserine residues and is important for recognition of apoptotic cells (84). Binding to TIM-3 on Th1 cells, however, does not account for all the function of one of its ligands, galectin-9, as it also binds to TIM-3 on DC. The putative function of galectin-9 in this context is to maintain peripheral tolerance to necrotic antigens (87).

The expression of TIM-3 in human DC is dependent upon the subset being examined. TIM-3 is expressed on CD11c+ PBMC (63). The level of its expression is high on the CD1c+ and CD141+ myeloid BDC subsets and MoDC, but low on pDCs (49, 88). This contrasts with a report suggesting high TIM-3 expression from tumor-associated MoDC in a murine colon cancer model and low expression on MoDC generated from healthy human donors (62).

The ability to take up and cross-present necrotic cells is an important mechanism by which DC sample tumor antigens. A positive regulatory role for TIM-3 on DC was initially reported as galectin-9 co-cultured with mature murine splenic DC increased TNF secretion (63). In contrast, other reports suggest that that antibody-mediated cross-linking of Tim-3 in bone marrow derived murine DC inhibited activation and maturation of DCs (89). TIM-3 blockade on CD103+ CD141+ DC enhances the effect of chemotherapy in a mouse model of breast cancer by improving CD8+ T cell effector cell infiltration in the presence of CXCL9 upregulation on DC (90), demonstrating a DC associated mechanism of TIM-3 targeting in addition to its role in T cells.

The function of TIM-3 may therefore be inhibiting or activating, depending on the immunological context. Blockade of TIM-3 in combination with blockade of CD28 family members such as PD-1 or CTLA-4 is rational as this reverts effector T cell function absent in exhausted cells (91). Blockade of TIM-3 on DC has direct effects which could be used to further enhance immune activation, e.g., by combining TIM-3 blockade with other treatments likely to increase tumor necrosis (e.g., chemotherapy) and uptake of necrotic antigens. Recent work suggests that anti-TIM-3 antibodies must bind to phosphatidylserine and CEACAM1 to have functional effects (92), therefore further work on defining important epitopes in in mAb blockade is required to optimize combination therapy with DC.

### Lymphocyte Activation Gene 3 Protein (LAG-3, CD223)

Lymphocyte activation gene 3 (LAG-3) is a receptor found on activated T and NK cells, which has sequence homology to CD4 (93). It binds to MHC Class II molecules abundantly expressed on DC. LAG-3 has a negative regulatory role in antigen-dependent T cell proliferation, particularly CD4+ T cells (93). LAG-3 is expressed in association with other immune checkpoint molecules in the tumor microenvironment, therefore chronic viral infection serves as a model for T cell exhaustion in tumor-associated lymphocytes (94). This has led to the development of antibodies against LAG-3 for clinical use in treatment of cancer alone or in combination with anti-PD-1 mAbs (Table 1).

Based on the inhibitory role of LAG-3 signaling in T cells, LAG-3: MHC-II interactions might be expected to be similarly inhibitory for DCs. However, experiments using soluble LAG-3-Ig reveal a stimulatory role in APC (95). In human MoDC, administration of soluble LAG-3-Ig fusion protein induces production of inflammatory cytokines (IL-8, MIP-1α/CCL3) and migratory chemokines (MDC/CCL22, TARC/CCL17 and CCR7) (65) which is not produced by an MHC II specific mAb, implicating the LAG3:MHC II interaction. LAG-3 expressed by activated T cells (96) directly stimulates MoDC to produce IL-12 and TNF without additional stimulation or co-stimulatory signals.

In addition to its role as a ligand for MHC Class II molecules on DC, LAG-3 is expressed by DC themselves. pDC are of particular importance in LAG-3 signaling. In humans, LAG-3+ pDCs in melanoma have been implicated in tumor-associated immunosuppression where tumor-associated MHC-II expression induces TLR-independent activation, production of inhibitory cytokines and recruitment of myeloid derived suppressor cells (MDSCs). Circulating human pDC are activated through LAG-3 in a TLR independent fashion with limited IFN-α and enhanced IL-6 production (97, 98), confirming a similar phenotype to tumor-associated suppressive pDC. Therefore, blockade of LAG-3 is a mechanism which has the potential to improve tumor control via either a DC or T cell mediated mechanism.

LAG-3 has direct effects on DC which promote their pro-inflammatory and migratory capacity. Whilst the development of anti-LAG-3 mAbs is of interest to enhance T cell responses, sLAG-3 is a preferable mechanism to target when used in combination with DC vaccination as an adjuvant. This is expected to promote DC stimulatory signals, migration and ultimately augment T cell function (Table 1).

## TNF Receptor Superfamily

Tumor necrosis factor receptor superfamily (TNFRSF) molecules are predominantly co-stimulatory receptors expressed on T cells. Their expression is regulated such that inducible co-stimulatory signals occur following the CD28-B7 interaction, sustaining T cell responses until attenuated by co-inhibitory molecules (Figure 2) (15). Co-stimulatory receptors include CD27 (TNFSR7), OX40 (CD134), 4-1BB (CD137), and CD40. With the exception of CD27 which is constitutively expressed, all of these receptors are induced hours or days after the initial APC: T cell interaction. The delayed kinetics of expression of TNSFR molecules suggests a role in sustaining immune responses and immune memory in contrast with the earlier expression of CD28 family receptors, making them attractive targets to help prolong anti-tumor responses.

Receptors in the TNFRSF fulfill the roles of DC priming, maintenance of memory cell pools and helper T cell pools (23, 72, 99).

The co-stimulatory role of TNFSF receptors is of translational interest as inadequate T cell priming has been implicated as a primary resistance mechanism to immune checkpoint inhibition (16). Numerous mAb agonists to TNFSRF receptors are currently being investigated in clinical trials in combination with anti PD-1 and anti-CTLA-4 mAbs, which modulate T cell co-inhibitory pathways. However, the potential for modulation of TNFSF ligands on DC as a strategy to enhance anti-tumor immunity is yet to be fully exploited. We review the key properties of each receptor that might be targeted in combination with DC therapy and the evidence for induction of their ligands on DC.

### CD40 (TNFSRF5)

CD40 is constitutively expressed on B cells, DCs, monocytes, and epithelial cells (100). It is unique amongst TNFRSF members due to its expression on DC rather than T cells. Its multiple functions include induction of B cell proliferation, isotype class switching and T cell help and is useful as a biomarker for disease progression in breast cancer, head and neck cancer and melanoma (66–68).

The ligand for CD40, CD40L (TRAP/T-BAM/CD154), is present on activated CD4+ T cells and is upregulated on human BDC by stimulation with anti CD40 antibody (100). Murine studies show that CD40L is upregulated on pDC stimulated via TLR-9 with CpG and is essential for licensing mDC to produce the pro-inflammatory cytokine, IL-12 (101). CD40 ligation on BDC results in production of IL-12, upregulation of the co-stimulatory molecules CD80 and CD86 and enhancement of CD8+ T cell priming (21).

CD40 fulfills the important roles of regulating T cell effector function, co-stimulatory molecule expression and Th1 polarization of naïve T cells via secretion of cytokines in addition to its use as a marker of DC activation. It is a unique TNFRSF molecule which modulates immune responses by production of cytokines which enhance T cell effector or B cell help functions (102) rather than via co-stimulation of antigenic signals through the TCR.

Anti CD40 mAb have direct anti-tumor efficacy which relies on activation of DC to induce anti-tumor T cell responses (103). CD40 mAb have been tested in clinical trials of B-cell lymphomas (104), pancreatic cancer (105) and other solid tumors as monotherapy (106), or in combination with chemotherapy (107). The mechanism of action for agonistic mAb is via licensing of DC to enhance anti-tumor T cell responses. The majority of CD40mAb are IgG1 and therefore bind to a wide range of FcγR and induce ADCC in target cells. Combination of DC vaccination with CD40 agonistic mAbs is expected to work in synergy generating tumor antigens for uptake by host DC via direct cytotoxicity as well as activation of those DC through CD40. This combination should be tested in a clinical trial of DC vaccination together with an IgG1 CD40 agonistic antibody to take advantage of host ADCC. However, the anti CD40 mAb, CP-870,893, which as an IgG2 has relatively poor activity via FcγR would not be predicted to be as effective.

### CD27 (TNFRSF7)

CD27 is co-stimulatory receptor which is expressed in humans on naïve and central memory T cells and progressively downregulated in effector memory cells (108). It contributes to the magnitude of both primary and memory responses to viral infection, acting either independently or together with CD28 to promote survival of primed CD8+ T cells (23). The agonistic CD27 mAb, varlimumab, promotes T-cell dependent tumor rejection in human CD27 transgenic mice (109), demonstrating that enhanced CD27 signaling can augment anti-tumor responses.

The ligand for CD27, CD70 (CD27L) is inducible on MoDC when they are exposed to TLR although the cytokine cocktail of PGE_2_, TNF, IL-1β, and IL-6 induces the strongest levels of CD70 (110). A similar effect is observed with human BDC (111). Myeloid DCs and pDCs stimulated with CD40L or PGE_2_ express CD70. Naïve CD4+ T cells stimulated with CD70+ MoDC produce Th1 and Th2 cytokines to a similar degree as CTLA-4/Fc, except that CD70mAbs prevented induction of IL-10.

The importance of CD27 in determining the magnitude of primary immune responses has implications for optimal priming of antigen-specific immune responses (23). As DC have the capacity to be loaded with antigens for therapeutic vaccination, the combination of antigen-loaded DC (Figure 1) enhanced with CD27 stimulating mAbs could have applications as a vehicle to enhance anti-tumor responses to defined tumor neo-antigens by exploiting the effects of the CD27 pathway on T cell priming.

In a murine model using OVA as a model antigen, CD27 agonistic mAbs preferentially generate antigen-specific CD8+ effector responses which are short-lived compared with those generated by 4-1BB activation, (112). These data suggest that CD27 ligation will stimulate a primary immune response that does not translate into effective T cell memory. An ideal combination of CD27 agonism with DC vaccination would be in boosting immune responses to low frequency antigens (e.g., tumor antigens) prior to co-stimulation through receptors with delayed expression and memory responses such as ICOS or 4-1BB. Experiments in the murine model showed that the combination of CD27 and 4-1BB activation abrogated the effect of 4-1BB activation alone on CD8+ T cell expansion.

In order to effectively translate this strategy into the clinic, further experiments with both low and high frequency antigens are required. The effect of CD27 activation in combination with DC vaccination is currently being tested in a phase I trial of a peptide vaccine plus the varlimumab for low grade glioma (NCT02924038).

### OX40 (CD134, TNFRSF4)

OX40 is a TNFRSF co-stimulatory receptor transiently expressed on activated CD4+ T cells within 12–24 h, with activation peaking after 2–3 days (30). Antigen-specific memory T cells re-express OX40 within 4 h following re-activation. It exerts direct functional effects on effector T cells via DC co-stimulation with its ligand, OX40L (CD252). Ligation of OX40 by CD252 promotes survival and expansion of CD4+ and CD8+ T cells and is essential for induction of Th2 responses (72). OX40L is expressed on CD4+ tumor infiltrating Tregs (71), where anti-OX40 mAb causes ADCC-dependent Treg depletion. The depletion of Tregs by OX40 mAb is reminiscent of a similar mechanism by CTLA-4. Agonistic OX40 mAbs have been evaluated extensively, showing anti-tumor activity in murine models of glioma, sarcoma, melanoma, and colon cancer (70). Therefore, OX40 represents a target of interest for clinical development of novel cancer immunotherapies.

The induction of OX40L on DC exploits the requirement for DC in induction of Th2 responses. Soluble CD40L induces OX40L and stimulates production of the pro-inflammatory cytokines, TNF, IL-1β, IL-6, and IL-12 (113). On BDC, OX40L is upregulated by sCD40L and associated with production of the cytokines IL-12p40 and IL-12p75 which are required for differentiation of naïve T cells into Th1 cells (113). Human mDCs upregulate OX40L when stimulated with thymic stromal lymphopoietin (114) whilst PGE_2_ similarly upregulates OX40L in CD1c+ mDCs (115). Furthermore, the CD141+ subset of mDC induces production of Th2 cytokines in CD4+ T cells in an OX40L dependent fashion (99).

The ability to preferentially induce CD4+ memory T cells capable of ensuring long-lasting antigen-specific responses through the modulation of signaling through OX40L on DC has strong translational potential in cancer immunotherapy. MoDC and CD1c+ and CD141+ BDC subsets upregulate OX40L when stimulated and produce pro-inflammatory cytokines necessary for T cell activation, supporting further *in vivo* studies to confirm that mature DC can be used to achieve anti-tumor responses in the same manner as agonistic OX40 mAbs.

OX40 activation skews T cell responses toward a Th2 phenotype rather than Th1, which is more commonly associated with anti-tumor cytotoxicity (116). OX40 agonists drive different responses depending on the cytokine milieu present at the time of administration (117). Administration of OX40 agonists during T cell priming drives Treg expansion and enhancement of experimental autoimmunity in contrast to what happens when administered at the time of priming in combination with a vaccine. Therefore, OX40 agonists administered in combination with DC vaccines should ideally be administered at the time of antigen priming. Further work defining the contribution of optimal *ex vivo* DC maturation factors to the cytokine milieu and therefore T cell phenotype is required before further translation.

### 4-1BB (CD137, TNFRSF9)

4-1BB (CD137/TNFRSF9) is a co-stimulatory receptor that exhibits inducible expression predominantly on activated CD8+ T cells which peaks at around 48 h. CD137 has multiple effects, including co-stimulation of CD4+ and CD8+ T cells, increase in T cell proliferation and IFN-γ production, and reduction of Treg infiltration in tumors (118, 119). Agonistic anti-41BB mAbs have shown anti-tumor efficacy in mice (120) mediated predominantly by CD8+ cells. This has led to clinical trials alone and in combination with T cell co-inhibitory blocking mAbs such as anti-PD-1 mAbs in advanced solid cancers (Table 1).

As in the case of OX40, DC enhance the anti-tumor immune responses generated by anti-4-1BB mAbs (120). Therefore, inducing the expression of the ligand for 4-1BB, 4-1BB-L during *ex vivo* priming of DC is of potential interest for translation of DC therapies in combination with 4-1BB receptor targeting.

In humans, 4-1BBL is expressed on MoDC activated with CD40L (121) whilst reverse signaling through 4-1BBL induces IL-12p70 secretion. This expression is functionally significant as co-culture with 4-1BB leads to cytokine production and co-stimulatory molecule up-regulation in DC. Furthermore, 4-1BB-L is able to induce proliferation and IFN-γ secretion by MoDC-stimulated antigen-specific T lymphocytes *in vitro* (121). However, the expression and function of 4-1BBL in BDC is yet to be fully addressed. The contribution of 4-1BB/4-1BB-L signaling, like that of CD27/CD70 and OX40/OX40L interactions described above, is in co-stimulation of effector T cell responses as well as polarization of T cell responses.

Lastly, 4-1BB activation induces memory T cells, with a particularly strong effects when administered during the priming phase (112). Combining 4-1BB activation with DC vaccination is therefore expected to act at the DC level by stimulating production of pro-inflammatory cytokines as well as improving the magnitude of memory responses elicited to specific antigens loaded onto DC during the priming phase. These factors are favorable for effective anti-cancer vaccination.

## Conclusion

DC based therapies hold great promise for application in cancer immunotherapy where the ability to induce specific, effective anti-tumor immune responses is required for successful clinical translation. Knowledge of the expression and functional consequences of immune checkpoint ligands and receptors on DC reviewed here is essential to identifying rational choices for combining DC vaccination with other therapies.

Immune checkpoint inhibitors have entered the mainstream for treatment of cancer. Successive clinical trials have shown improvement in clinical response rates with anti-CTLA-4 mAbs then anti-PD-1 mAbs. Combined targeting of these pathways has improved response rates, but many patients exhibit resistance to treatment. Overcoming resistance to immune checkpoint inhibitors due to poorly immunogenic tumors would address a key unmet medical need. DC vaccination circumvents the problem of poorly immunogenic tumors by stimulating specific immune responses to tumor antigens in naïve T cells. This approach alone has had limited success in the clinic despite inducing measurable immune responses (122), indicating that an anti-tumor response itself does not always overcome tumor-associated immunosuppression. Immune checkpoint inhibitors are predicted to work in synergy with DC vaccination by acting directly on DC to enhance their function in addition to their known ability to re-invigorate exhausted T cells.

Combinations of DC vaccination with different checkpoint inhibitors enhance antigenic stimulation at different stages of the immune response. For tumors which are immunologically “cold” (123) due to a low tumor mutational burden such as pancreatic cancer, CD27 agonistic mAbs in combination with tumor-antigen loaded DC could be used to expand the T cell repertoire. Soluble LAG-3 promotes cellular maturation and migration, resulting in further improvement of anti-tumor immunity against these antigens (Figure 1). Immune stimulatory therapies such as oncolytic viruses have been shown to improve responses to anti-PD-1 mAbs by enhancing T lymphocyte infiltration into the tumor microenvironment (124), therefore combining DC vaccination with targeting of CD27 and LAG-3 is predicted to have a similar effect via enhanced antigen-specific priming with CD27 agonism or enhanced DC migration and antigen presentation with LAG-3. For tumors that are rendered poorly immunogenic by a high Treg burden, the combination of DC vaccination with Treg depleting anti-OX40 mAbs is expected to have anti-tumor activity. The use of mAbs against TIM-3 is ideal for combination with therapies that will increase necrotic antigen production such as concurrent chemotherapy and radiotherapy for rectal or head and neck cancers. TIM-3 blockade on DC administered in combination with radiotherapy could enhance uptake of tumor-associated antigens from necrotic cells and therefore immune responses to these antigens. Agonistic CD40 mAb, particularly IgG1 isotype, is ideal for combination therapy due to their ability to induce ADCC, providing tumor antigens for subsequent uptake by DC. This is predicted to have particularly strong synergy in CD40 expressing cancers (Table 1).

Immune checkpoint inhibition affects the nature of T cell responses. CD40 or 4-1BB agonistic Abs polarize T cell responses toward a Th1 phenotype characteristic of cytotoxic responses whilst ICOS agonists promote class switching and memory responses. Clinical studies have demonstrated that alterations of the Th1/Th2 ratio is associated with defects in cellular immunity seen in glioblastoma, lung cancer and non-Hodgkin lymphoma (125). This warrants further investigation in combination with DC vaccination to ensure control over the presentation of tumor neo-antigens to Th1 cells. The unique Th1 polarizing effect of DC which have been stimulated with CD40L means that CD40L mAb combinations with DC vaccination could act as a backbone for DC combination therapies in the same way that anti-PD-1 mAbs are emerging as a backbone of combination immune checkpoint inhibitor combinations.

Effective combination treatment may be achieved by agonistic mAbs or concomitant induction of ligands for the co-stimulatory TNFRSF receptors CD27, OX40L, and 4-1BB as well as the Ig superfamily receptor ICOS on DC during the *ex vivo* maturation of DC for vaccination. However, it remains to be seen whether DC driven effects on T cells will effectively augment the effect of agonistic mAbs *in vivo*. The precise spatiotemporal sequence of signaling events *in vivo* must be determined at a cellular level and in draining lymph nodes for DC vaccination to be translated. Upregulation of CD70, OX40L and 4-1BB-L on DC may be achieved by common stimuli such as CD40L which makes translation of DC therapy an attractive strategy to simultaneously activate multiple co-stimulatory pathways. It is likely that DC vaccination, if applied in the clinic, will result in conditions which favor activation of these pathways which could be further augmented with TNFRSF receptor agonistic mAbs given at times which augment their expression patterns (Figure 1) or combined with blocking mAbs against co-inhibitory receptors such as CTLA-4 or PD-1. Together with CD40 receptor ligation, activation of co-stimulatory pathways with DC vaccination and attenuation of co-inhibitory pathways with blocking mAbs is predicted to synergistically enhance antigen-specific immunity and licensing of cytotoxic T cell responses at all stages of the immune response, holding great promise for rational development of cancer immunotherapy.

## Author Contributions

Writing by BK, HB, and JK. Review and Figures by PS. Concept by PF and GC.

### Conflict of Interest Statement

GC is a Director of DendroCyte Biotech Pty Ltd. The remaining authors declare that the research was conducted in the absence of any commercial or financial relationships that could be construed as a potential conflict of interest.

## Abbreviations

DC: Dendritic Cells
BDC: Blood Dendritic Cells
MoDC: Monocyte-Derived Dendritic Cells
pDC: Plasmacytoid Dendritic Cells
mDC: Myeloid Dendritic Cells
PBMC: Peripheral Blood Mononuclear Cells
mAb: Monoclonal Antibody
MLR: Mixed leucocyte Reaction
Treg: Regulatory T Cell.
